# B7-H3 and ICAM-1 are potentially therapeutic targets for thyroid carcinoma

**DOI:** 10.1186/s13000-024-01504-2

**Published:** 2024-06-10

**Authors:** Pengtao Song, Yongcan Xu, Guochao Ye

**Affiliations:** 1 Department of General Surgery, Fifth School of Clinical Medicine of Zhejiang, Huzhou Central Hospital, Chinese Medical University, Huzhou, People’s Republic of China; 2 Department of General Surgery, Huzhou Central Hospital, Affiliated Central Hospital, Huzhou University, Huzhou, 313000 People’s Republic of China; 3 Department of Pathology, Fifth School of Clinical Medicine of Zhejiang, Huzhou Central Hospital, Chinese Medical University, Huzhou, People’s Republic of China; 4 Department of Pathology, Huzhou Central Hospital, Affiliated Central Hospital Huzhou University, Huzhou, People’s Republic of China

**Keywords:** B7-H3, ICAM-1, Thyroid cancer

## Abstract

Although most differentiated thyroid carcinoma has a clinically favorable prognosis, some of specific types of thyroid cancer (such as anaplastic thyroid carcinoma and advanced papillary thyroid carcinoma) show fatal outcomes and require novel treatments. Immunotherapy is a promising avenue for the treatment of advanced thyroid carcinoma. B7-H3 (B7 homolog 3 protein) and ICAM-1 (intercellular adhesion molecule 1), as two important immune checkpoints (ICPs), is becoming hopeful target spots for immunotherapy. A growing amount of evidence has suggested that B7-H3 and ICAM-1 are upregulated in papillary thyroid carcinoma. However, their expression level in specific types of thyroid cancer remains largely unclear. In the present study, we explored the expression level of B7-H3 and ICAM-1 in different types of thyroid carcinoma. In the groups of the TCGA cohort, both B7-H3 and ICAM-1 mRNA were highly expressed in thyroid carcinoma. Furthermore, the patients with Stage2, 61-80y, Follicular thyroid papillary carcinoma and N0 had lower B7-H3 and ICAM-1 mRNA expression. In the groups of our cohort, PTCs and ATCs showed frequently moderate to strong expression of B7-H3 and ICAM-1 protein expression. The significant relevance of B7-H3 staining score with ICAM-1 staining score was observed in TCGA database and our cohort, which might open avenues for the combination therapy in advanced thyroid cancer.

## Introduction

Thyroid cancer is the most frequent type of endocrine system malignancy and the incidence continues to rise worldwide [1]. It is usually categorised into three broad histological categories [2]: (1) Differentiated thyroid cancer, which originates from thyroid follicular epithelial cells, encompassing papillary, oncocytic and follicular thyroid cancer; (2) Anaplastic thyroid cancer (ATC), which often arises from and can coexist with differentiated thyroid cancer; (3) Medullary thyroid cancer (MTC), which originates in the parafollicular neuroendocrine cells of the thyroid; The diverse heterogeneity of thyroid cancers results in distinct prognoses. The most prevalent subtype of thyroid cancer, papillary thyroid cancer (PTC), typically presents as a slowly progressing tumor and carries the most favorable overall prognosis [3]. In contrast, a small proportion of thyroid carcinomas, such as poorly-differentiated PTC (PDPTC), MTC, and ATC, exhibit high aggressiveness and even lethality [4]. Conventional treatments for thyroid cancer include surgical intervention, radioactive iodine (RAI) therapy, and thyroid stimulating hormone suppression therapy, either alone or in combination [5]. Furthermore, targeted therapies and antiangiogenic multikinase inhibitors are approved for thyroid cancer recently [6]. However, there is a paucity of effective treatment options available for patients with advanced thyroid cancer.

Previous studies have postulated that the distinct prognosis is a result of intricate interactions between tumor cells and their microenvironment [7]. Indeed, immunotherapy utilizing immune checkpoint inhibitors targeting PD1, PD-L1, and CTLA4 has emerged as a well-established option for cancer treatment in recent decades [8]. Similarly, active trials of immunotherapy are underway in patients with thyroid cancer and have shed light on the potential of this treatment approach for advanced cases. In the KEYNOTE-028 trial, two patients with advanced PTC who tested positive for PD-L1 achieved a partial response following treatment with pembrolizumab (an anti-PD1 therapy) [9]. Additionally, single-agent spartalizumab (anti-PD1) treatment demonstrated an ORR of 19% (8 out of 42 patients) in progressive ATC patients [10]. Although immunotherapy cannot currently replace the standard of care, immune-based approaches remain a promising avenue for treatment.

In this study, we analyzed the expression level of two immune checkpoints (ICPs) B7H3 (B7 homolog 3 protein) and ICAM-1 (intercellular adhesion molecule 1) as well as their correlation between clinic-pathological features in thyroid cancer. Moreover, we initially evaluate the correlations between the levels of B7-H3 and ICAM1 in thyroid cancer.

## Materials and methods

### Analyses of the UALCAN databases and GSE213647

UALCAN (https://ualcan.path.uab.edu/analysis.html) is an integrated cancer data analysis platform based on the TCGA databases [11]. In the present study, the UALCAN website is used to explore the expression levels of B7-H3 and ICAM-1 in thyroid cancer and normal thyroid tissues. Additionally, the correlation between B7-H3 and ICAM-1 in thyroid cancer is evaluated in GSE213647.

### Tissue samples collection and ethics statement

Tissue arrays (HThyCan060PT01) containing 28 patients who underwent surgical resection for thyroid cancer were obtained from Shanghai Outdo Biotech. The study design was approved by the Institutional Review Board of Huzhou central Hospital/Shanghai Qutdo Biotech Company Ethics Committee. All pathologic specimens were independently certified by two pathologists. Stage classification was assessed according to the tumor-node metastasis classification system (8th AJCC). The clinicopathologic characteristics of the patients are summarized in Table 1.

**Table 1:** Baseline characteristics

	All samples (*n*=28)
Age, Years	56.11 (11.31)
Sex	
Male	9 (32.14%)
Female	19 (67.86%)
Pathological Type	
PTC	18 (64.29%)
MTC	6 (21.43%)
FTC	2 (7.14%)
ATC	2 (7.14%)
Differentiation	
Well	20 (71.43%)
Moderate	6 (21.43%)
Poor	2 (7.14%)
Tumor Size	
T1	11 (39.29%)
T2	15 (53.57%)
T3	1 (3.57%)
T4	1 (3.57%)
Lymphatic Metastasis	
N0	25 (89.29%)
N1	3 (10.71%)
Distant Metastasis	
M0	28 (100%)
M1	0 (0%)

Data are mean (SD) or n (%)

### Construction of tissue microarrays (TMAs)

Twenty-eight cases of thyroid cancer tissues and paired non-neoplastic thyroid tissue were confirmed by reviewing hematoxylin and eosin (H&E) stained slides. To construct TMAs, one representative formalin-fixed paraffin-embedded archival block were selected for each case. Then, 4 mm thick tissue cores were extracted from individual paraffin blocks and subsequently re-arranged into TMA blocks. The TMA blocks were incubated at 60 ℃ for about 30 min and cooled at normal temperature. Lastly, TMA blocks were cut into 4 µm thick sections and subjected to the usual immunohistochemistry protocol.

### Immunohistochemistry (IHC) procedure

Tissue Sects. (4 µm) were cut from TMA blocks and used for IHC. Deparaffinization of these sections was performed with xylene for 20 min and a graded series of decreasing alcohol series, and then washed with phosphate-buffered saline (PBS). Next, endogenous peroxidase activity was blocked with 0.3% hydrogen peroxidase in methanol at 37 ℃ for 30 min, and 5% goat serum was applied to block the nonspecific binding. IHC staining was performed by using primary antibodies in a humidified chamber at 4 ℃ overnight. The primary antibodies were an antibody against B7-H3 (1:10,000 dilution, Cat. no ab219648, Abcam, Cambridge, UK) and an antibody against ICAM-1 (1:500 dilution, Cat. no 67836S, CST, Beverly, United States). Subsequently, the sections were washed with PBS and probed with secondary antibodies for 2 h. Then, the sections were treated with Vectastain ABC reagent for 30 min and visualized with the DAB chromogen for 10 min. For negative control the primary antibody was omitted.

### Evaluation of immunohistochemical staining

A total of 56 TMA points were retained for further analysis. To quantify B7-H3 and ICAM-1 protein expression, all stained points were separately evaluated by two senior pathologists. Both the staining extensity and intensity were assessed in randomly selected five representative fields of vision. According to the percentage of positive tumor cells, B7-H3 and ICAM-1 protein expression were further stratified into negative (< 1%), low (1% ~ 49%), and high (≥ 50%).

### Statistical analysis

SPSS 19.0 software (IBM, Chicago, IL, USA) was used for all data analyses and differences were considered to be statistically significant differences at *P* < 0.05. All results were expressed as the mean ± standard deviation (S.D.). The chi-square test and Student's t-test (two-tailed) were used to evaluate differences between groups.

## Results

### The expression level of B7-H3 and ICAM-1 mRNA in thyroid cancer

**Fig. 1:**
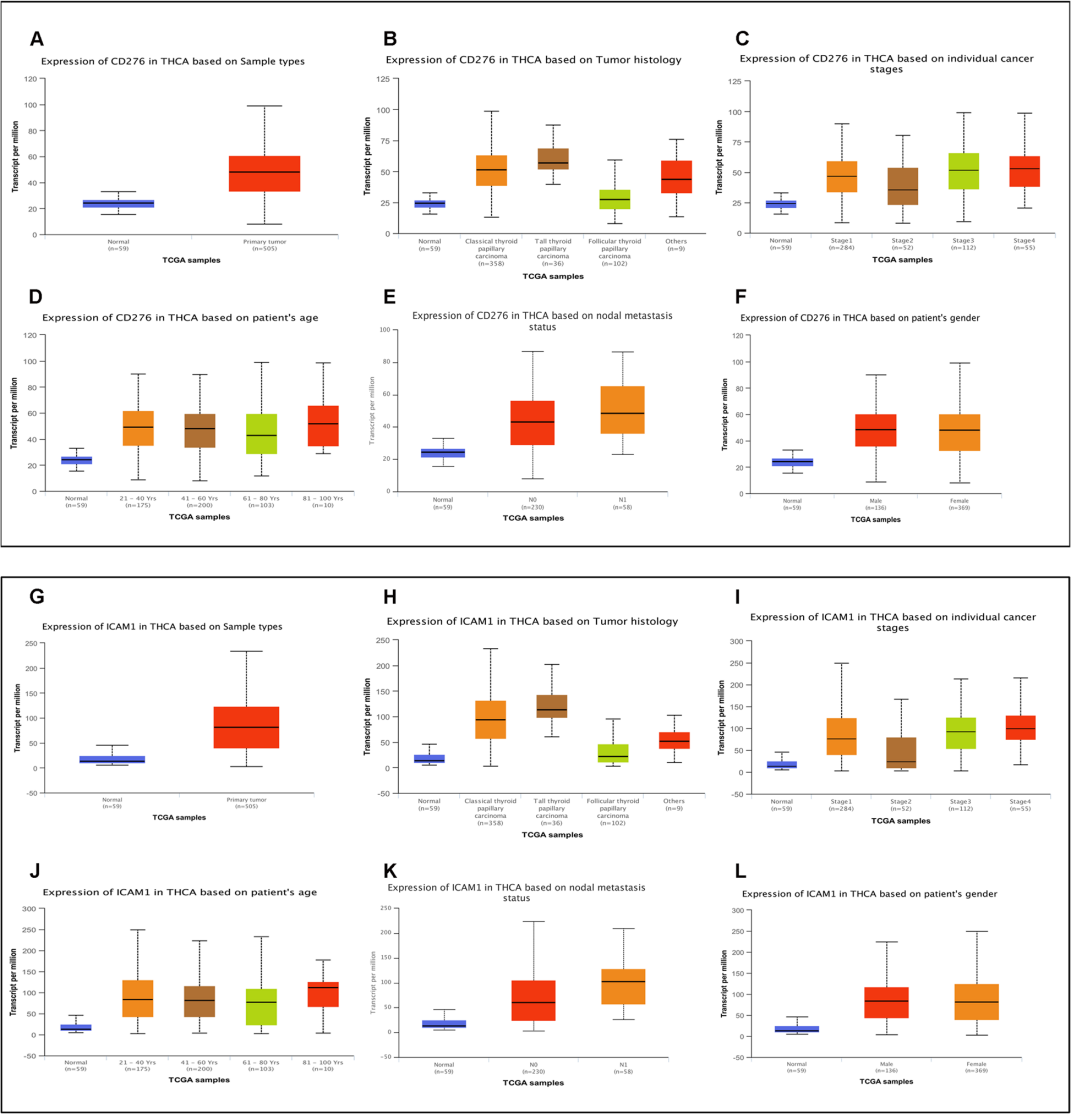
Expression levels of B7-H3 and ICAM-1 mRNA in thyroid carcinoma (THCA) in the TCGA cohort. **A**. B7-H3 mRNA expression between normal thyroid tissue samples (*n* = 59) and thyroid cancer tissue samples (*n* = 505). **B**. B7-H3 mRNA expression of tumor tissues with different tumor histology. **C**. B7-H3 mRNA expression of tumor tissues with different tumor stages. **D**. B7-H3 mRNA expression of tumor tissues with different patient's age. **E**. B7-H3 mRNA expression of tumor tissues with different nodal metastasis status. **F**. B7-H3 mRNA expression of tumor tissues with patient's gender. **G**. ICAM-1 mRNA expression between normal thyroid tissue samples (*n* = 59) and thyroid cancer tissue samples (*n* = 505). **H**. ICAM-1 mRNA expression of tumor tissues with different tumor histology. **I**. ICAM-1 mRNA expression of tumor tissues with different tumor stages. **J**. ICAM-1 mRNA expression of tumor tissues with different patient's age. **K**. ICAM-1 mRNA expression of tumor tissues with different nodal metastasis status. **L**. ICAM-1 mRNA expression of tumor tissues with patient's gender

As previously described, B7-H3 (CD276) and ICAM-1 (CD54) has been extensively studied in various cancers, including lung cancer, breast cancer and so on (1,2). In the TCGA databases, there was a significant difference in B7-H3 (CD276) expression between normal thyroid tissue samples (*n* = 59) and thyroid cancer tissue samples (*n* = 505) (*p* < 0.001, Fig. 1A), B7-H3 (CD276) is highly expressed in tumor tissues. Furthermore, the expression level of B7H3 (CD276) was significantly different in histology, stages, patient's age and nodal metastasis status (Fig. 1B-E). Statistical analysis indicated that the patients with Stage2, 61-80y, Follicular thyroid papillary carcinoma and N0 had lower B7-H3 (CD276) mRNA expression. there was no significant association between B7-H3 (CD276) expression and patient's gender (Fig. 1F). Similar to B7-H3, the same result was also obtained in the analysis of ICAM-1(CD54) mRNA and thyroid cancer tissue samples (Fig. 1G-L). These data indicated that the level of B7-H3 (CD276) and ICAM-1(CD54) mRNA are up-regulated in thyroid cancer tissues.

### Patient characteristics in our cohort

A total of 28 patients with thyroid cancer were enrolled, including 18 PTCs, 6 MTCs, 2 FTCs, 2 ATCs (among them one is SCC). Table 1 summarizes the basic clinical data. In the overall cohort, 9 were men and 19 were women. The mean age at diagnosis was 56 years. Moreover, 71.4% (20/28) cases had well differentiated tumor and 35.7% (10/28) patients had small tumor size (maximum diameter < 2 cm). Among these patients, 3 patients showed signs of lymph node metastasis (N1) and no patients showed distant metastasis.

### Immunohistochemical (IHC) staining of B7-H3 and ICAM-1 in thyroid cancer

**Fig. 2:**
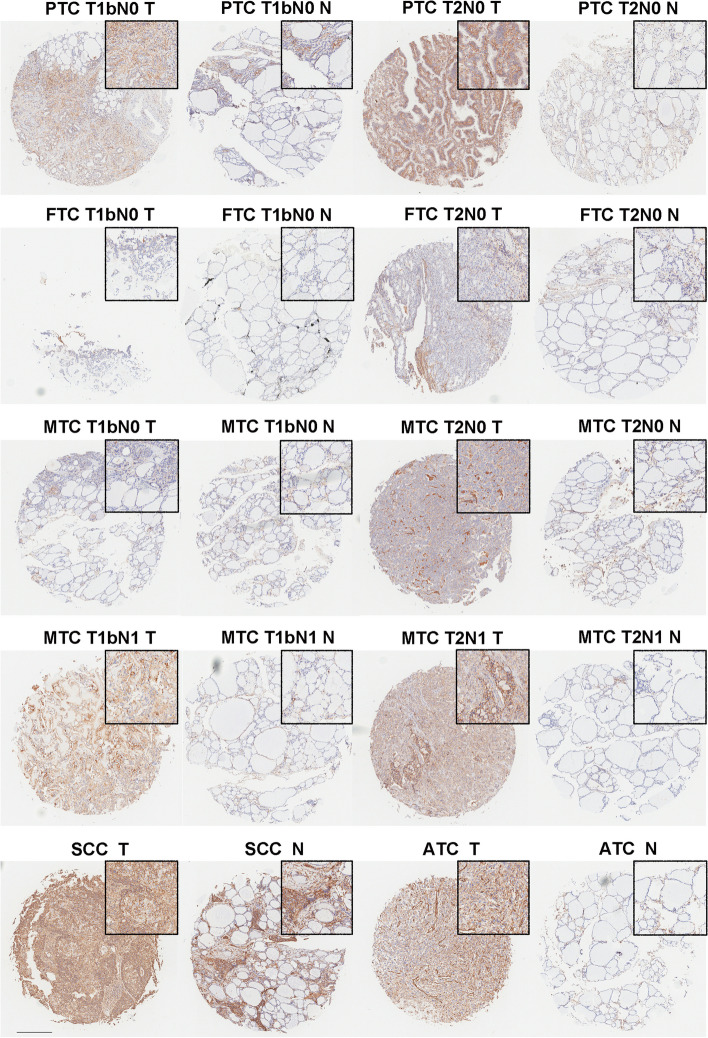
Representative photomicrographs of B7-H3 immunohistochemical expression in different types and stages of thyroid carcinoma

**Fig. 3:**
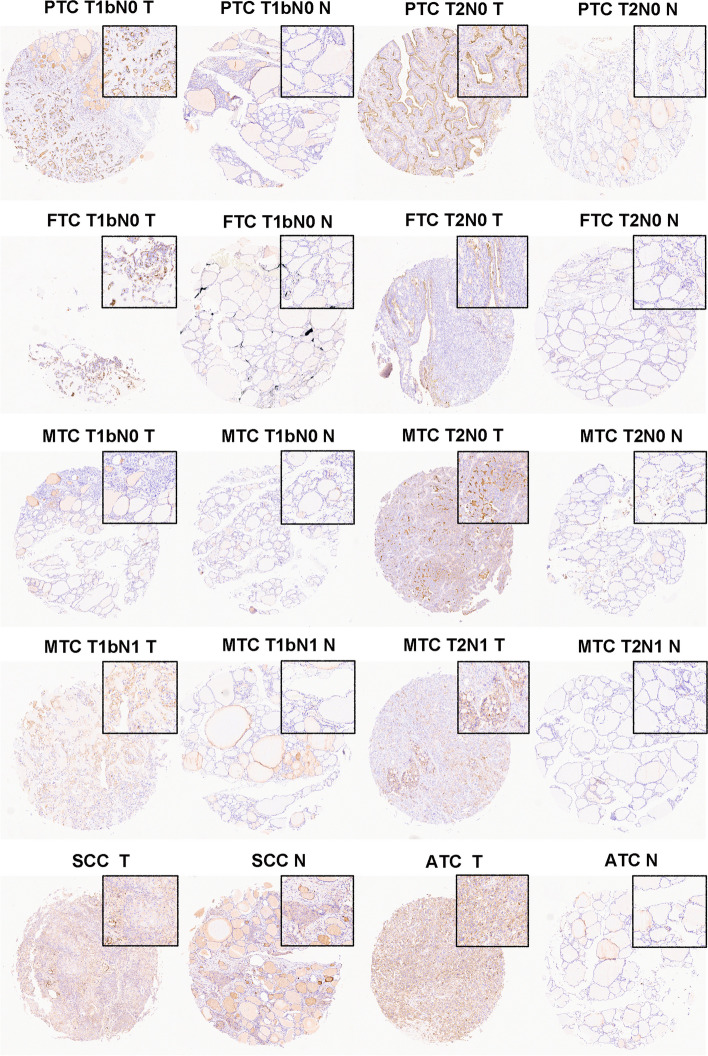
Representative photomicrographs of ICAM-1 immunohistochemical expression in different types and stages of thyroid carcinoma

**Fig. 4:**
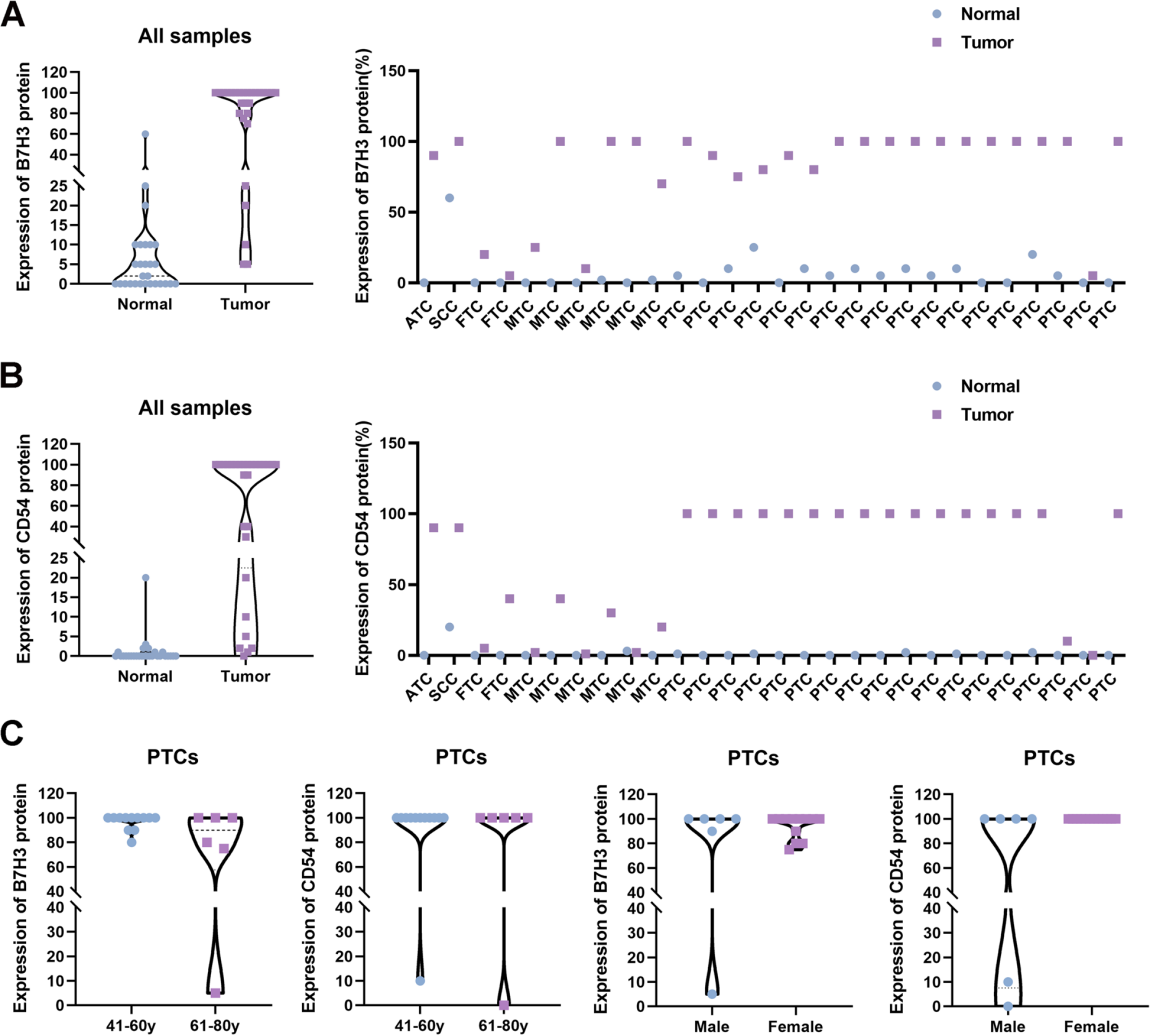
Expression levels of B7-H3 and ICAM-1 protein in thyroid carcinoma in our cohort. **A**. Immunohistochemical expression of B7-H3 in thyroid carcinomas (*n* = 28) and paired adjacent normal tissue (*n* = 28) (left). Immunohistochemical expression of B7-H3 in each thyroid carcinoma sample(right). **B**. Immunohistochemical expression of ICAM-1 in thyroid carcinomas (*n* = 28) and paired adjacent normal tissue (*n* = 28) (left). Immunohistochemical expression of ICAM-1 in each thyroid carcinoma sample(right). **C**. The correlation between the clinical features of PTCs (patient's age and gender) and B7-H3 (CD276) or ICAM-1 (CD54) protein expression

To explore the protein level of B7-H3 (CD276) and ICAM-1(CD54) in thyroid cancer, IHC studies were conducted in samples of the 28 thyroid cancer cases. Both B7-H3 (CD276) and ICAM-1(CD54) staining were mainly localized to the cytoplasmic /membranous of tumor cells and the representative regions of IHC are shown in Figs. 2 and 3. Via IHC, B7-H3 (CD276) protein expression was found in 28 of 28 thyroid cancers (100%) and had a significantly higher expression of B7-H3 (CD276) expression in tumor tissues than in normal tissues (Fig. 2B). Among the positive cases, MTCs and FTCs exhibited relatively weaker expression of B7-H3 (CD276). While, PTCs and ATCs showed frequently moderate to strong expression of B7-H3 (CD276). Moreover, compared with normal tissues, ICAM-1(CD54) was also increased in thyroid cancer tissues (Fig. 3B). The results showed that ICAM-1 (CD54) protein expression was detected in 27 thyroid cancer tissues, including low expression levels in 9 samples (33.3%) and high expression levels in 18 samples (66.7%). Overall, these data suggest that the expression of B7-H3 (CD276) and ICAM-1 (CD54) were up-regulated in thyroid cancer tissues (Fig. 4A and B).

Furthermore, the correlation between the clinical features of PTCs (patient's age and gender) and B7-H3 (CD276) or ICAM-1 (CD54) protein expression were analyzed. We found that the patients with 61-80y had lower protein expression of both B7-H3 (CD276) and ICAM-1 (CD54). However, the lack of statistical difference between the two groups (41-60y and 61-80y) might be due to the small sample size and semi-quantitatively score (*p* = 0.079 and *p* = 0.567). However, there was no significant association between B7-H3 (CD276) or ICAM-1 (CD54) expression and patient's gender (Fig. 4C).

### Relation between level of B7-H3 and ICAM-1 expression

**Fig. 5:**
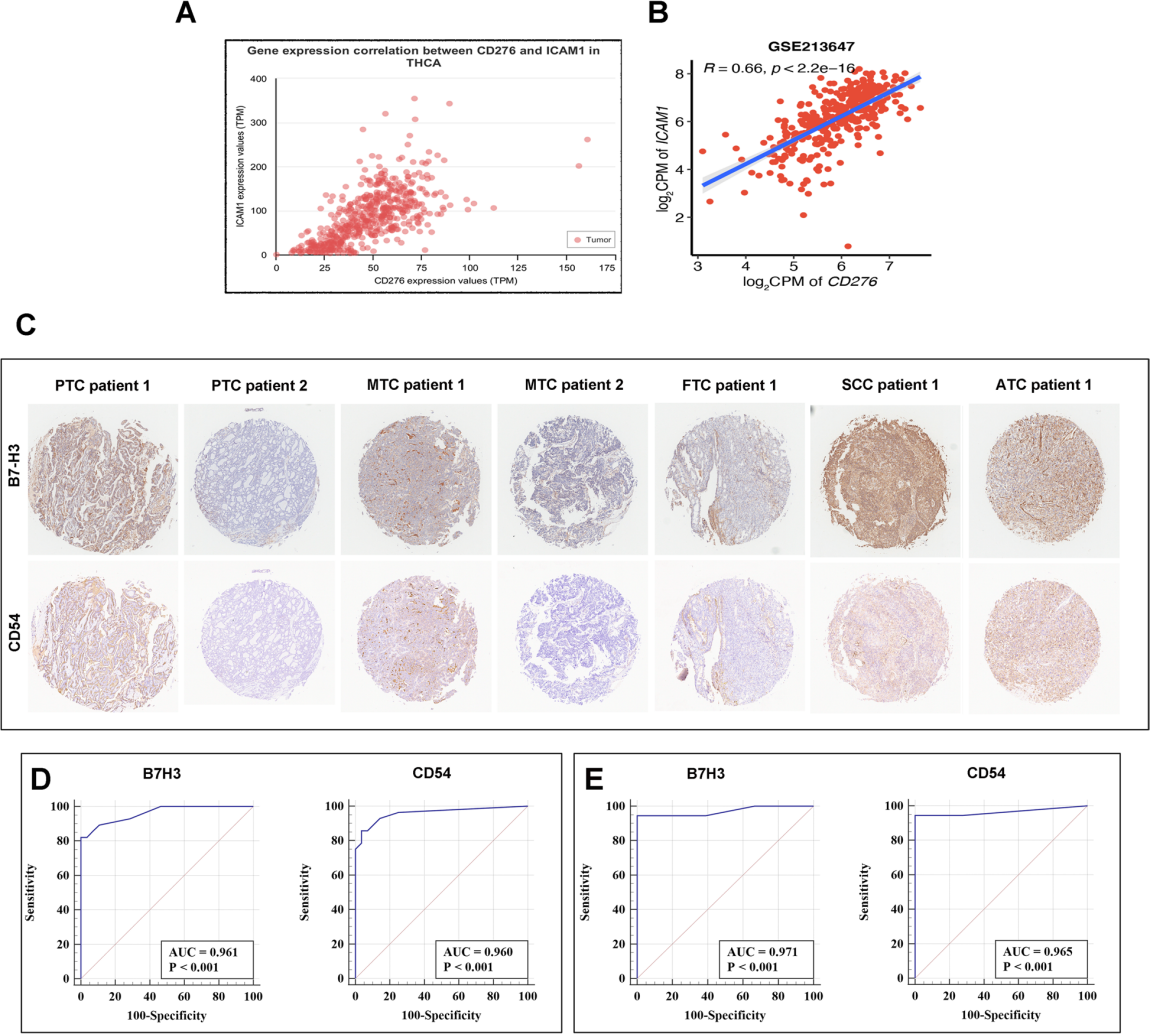
Correlations between B7-H3 and ICAM-1 and the diagnostic value of B7-H3 and ICAM-1 in thyroid cancer. **A**. Correlations between B7-H3 and ICAM-1 mRNA in different types of thyroid carcinoma in TCGA cohort. **B**. Correlations between B7-H3 and ICAM-1 mRNA in different types of thyroid carcinoma in GSE213647. **C**. Correlations between B7-H3 and ICAM-1 protein in different types of thyroid carcinoma in our cohort. **D**. ROC curve showing the diagnostic significance of B7-H3 (left) and ICAM-1 (right) level in thyroid cancer. **E**. ROC curve showing the diagnostic significance of B7-H3 (left) and ICAM-1 (right) level in PTC

Besides, we searched the TCGA database and Gene Expression Omnibus database (GEO) to further investigate the correlation between B7-H3 and ICAM-1 expression. The result showed B7-H3 mRNA level was positively correlated with ICAM-1 mRNA level in THCA (thyroid carcinoma database in TCGA) and GSE213647 (thyroid carcinoma database in GEO) (Fig. 5A and B). Analogously, we observed significant relevance of B7H3 staining score with ICAM-1 staining score and HR of them co-expression was 0.6423 (95% CI: 0.3540–0.8191, *p* = 0.0002). Representative regions of B7H3 and ICAM-1 staining are depicted in Fig. 5C.

### Diagnostic value of B7-H3 and ICAM-1 in thyroid cancer

To validate the potential diagnostic utility of B7-H3 and ICAM-1, we firstly performed receiver operating curve (ROC) analysis in thyroid cancer patients. The area under the curve values (AUCs) were 0.961 for B7H3(CD276) and 0.960 for ICAM-1(CD54) (Fig. 5D). The sensitivities of B7-H3 and ICAM-1 were 82.1% and 85.7%. While, the specificities of B7-H3 and ICAM-1 were 100.0% and 96.4%. Furthermore, we also validated the potential diagnostic utility of B7-H3 and ICAM-1 in PTC patients. Analogously, the area under the curve values (AUCs) were 0.971 for B7H3(CD276) and 0.965 for ICAM-1(CD54) (Fig. 5E). The sensitivities and specificities of B7-H3 and ICAM-1 were both 94.4% and 100.0%.

## Discussion

It has been proved that the successes of immune checkpoint (ICP) inhibitors have reinvigorated the field of anti-cancer therapy [12, 13]. Recent scientific advances in our understanding of immune microenvironment involved in thyroid cancer contribute to the identification of new efficacy biomarkers and novel immunotherapeutic approaches. Luo et al. investigated potential clinical significance of ICP in patients with advanced thyroid cancer [14]. They observed that PD-L1 is related to the OS of ATC. Meanwhile, B7-H3 and VISTA are two important prognostic biomarkers in advanced PTC. Indeed, for the patients with advanced thyroid cancer that are refractory to traditional therapies, immunotherapies had started to become promising therapeutic options. In addition to immune checkpoints inhibitor, adoptive cell therapy is another area of personalized cancer treatment. The thyroid-stimulating hormone receptor (TSHR)-specific CAR T cells diminished tumors of a DTC xenograft mouse model [15].

B7-H3, also known as CD276, is a member of the B7-CD28 immunoregulatory protein superfamily [16]. It was recognized as a co-stimulatory or co-inhibitory molecule which plays a dual role in the immune reactions [17]. Andrei I. Chapova and colleagues found that B7-H3 could costimulate proliferation of T cells and selectively stimulate interferon γ (IFN-γ) production [18]. Moreover, in a study among colon cancer, the antitumor immunity was established by adenoviral B7-H3 transfer [19]. Critically, the inhibitory effects of B7-H3 on immune microenvironment was discovered. Emerging studies have demonstrated that B7-H3 influence both the immune response and tumor behavior through different signaling pathways, and B7-H3 level is associated with poor prognosis [20]. In order to explore the immune and clinical features of B7-H3 in PTC patients, Zhao et al. performed large-scale analyses [21]. Their observations revealed that the PTC B7-H3 status might serve as a novel predictive biomarker for disease recurrence for patients who undergo an aggressive disease course. Recently, numerous of anti-B7-H3 approaches have been investigated in preclinical or clinical trials, such as a humanized mAb targeting B7-H3 (Enoblituzumab), anti-B7-H3 bispecific antibody and B7-H3-specific CAR-T cells [22]. Researchers focus on the antitumor ability of the different approaches against B7H3 in various B7-H3 positive tumors [22]. In our study, we obtained that B7-H3 was observably increased in several types of thyroid cancer tissues, such as PTCs, ATCs. Our result is crucial and may help to provide evidence for therapeutic application of anti-B7-H3 agents in different types of thyroid cancer patients.

ICAM-1, also known as CD54, is a transmembrane glycoprotein which is present primarily on endothelial cells and leukocytes [23]. It plays a crucial role in stabilizing cell–cell interactions and promoting leukocyte endothelial transmigration [24]. More recently, it is increasingly clear that ICAM-1 is over-expressed on the surface of various types of tumors [25]. ICAM-1 can contribute to tumor growth, metastasis and angiogenesis [26]. Thus, a better understanding of its vital roles will provide therapeutic strategies in cancer treatment. A growing amount of evidence has suggested that ICAM-1 is critical regulator of thyroid cancer. D. Buitrago et al. have previously reported that ICAM-1 is upregulated in aggressive PTC [27]. Besides, Zhang et al. also found that ICAM-1 expression is increased in PTC and in Hashimoto's thyroiditis (HT) with PTC-like nuclear alterations [28]. Similarly, our results presented that PTCs and ATCs showed frequently moderate to strong expression of ICAM-1 protein expression Notably, Yogindra Vedvyas and co-worker not only have shown a significant relationship between ICAM-1 overexpression and malignancy in thyroid cancer, but also have pioneered the use of ICAM-1 targeted CAR T cells as a novel treatment modality [29]. Subsequent studies found that PD1 blockade could reverse exhausted ICAM1-CAR T cells in ATC mouse model, which implicates that combination therapy of ICAM1-targeting CAR T cells and PD1/PD-L1 blockade has a potential to control advanced thyroid cancers [30].

In this research, we are the first to show the co-expression of ICAM1 and B7H3 mRNA as well as their associations between clinic-pathological features in thyroid cancers. Furthermore, the correlation between ICAM1 and B7H3 were also evaluated. If the interacting mechanism between ICAM1 and B7H3 can be full identified, we might open avenues for the combination therapy in advanced thyroid cancer (Fig. 5).

## Data Availability

No datasets were generated or analysed during the current study.
